# Treatment patterns and outcomes in elderly patients with newly diagnosed multiple myeloma: results from the Connect^®^ MM Registry

**DOI:** 10.1038/s41408-021-00524-1

**Published:** 2021-07-23

**Authors:** Hans C. Lee, Sikander Ailawadhi, Cristina J. Gasparetto, Sundar Jagannath, Robert M. Rifkin, Brian G. M. Durie, Mohit Narang, Howard R. Terebelo, Kathleen Toomey, James W. Hardin, Lynne Wagner, James L. Omel, Mazaher Dhalla, Liang Liu, Prashant Joshi, Rafat Abonour, Connect® MM Registry, Rafat Abonour

**Affiliations:** 1 The University of Texas MD Anderson Cancer Center, Houston, TX USA; 2 Mayo Clinic, Jacksonville, FL USA; 3 Duke University Medical Center, Durham, NC USA; 4 Mount Sinai Hospital, New York, NY USA; 5 Rocky Mountain Cancer Centers US Oncology Research, Denver, CO USA; 6 Cedars-Sinai Medical Center, Los Angeles, CA USA; 7 Maryland Oncology Hematology, US Oncology Research, Columbia, MD USA; 8 Providence Cancer Institute, Southfield, MI USA; 9 The Steeplechase Cancer Center, Somerville, NJ USA; 10 University of South Carolina, Columbia, SC USA; 11 Wake Forest School of Medicine, Winston-Salem, NC USA; 12 Myeloma Research Advocate/Advisor, Grand Island, NE USA; 13 Bristol Myers Squibb, Princeton, NJ USA; 14 Indiana University, Indianapolis, IN USA

**Keywords:** Haematological diseases, Therapeutics

**Dear Editors**,

Multiple myeloma (MM) is a common disease among the elderly with a median age of 70 years at diagnosis and many patients diagnosed at ≥75 (~30%) and ≥85 years (~10%) [1, 2]. The incidence and prevalence of newly diagnosed MM (NDMM) and relapsed/refractory MM among elderly patients are expected to increase over time due to lengthening life expectancy [3]. Despite the frequency of MM diagnoses, older patients are underrepresented in clinical trials due to eligibility criteria or physician, patient, or caregiver barriers, resulting in a lack of data on their treatment patterns and outcomes [4].

Retrospective analyses have demonstrated the potential benefits of actively treating elderly patients with MM, particularly with novel agents [5–7]. However, retrospective data are less robust than those from prospective studies [8], and few studies focus on treatment patterns or characterization of elderly patients with MM. A real-world, prospective, longitudinal study in this population would thus address the lack of clinical trial data and limitations of retrospective studies. The Connect^®^ MM Registry (NCT01081028) is a large, US, multicenter (84% community sites), prospective observational cohort study. A descriptive analysis of treatment patterns and survival outcomes in elderly patients (≥75 years old) enrolled in the Registry is presented here.

The Connect MM Registry has previously been described in detail and is outlined in the Supplemental Methods [9]. Briefly, patients aged ≥18 years with symptomatic MM [10] diagnosed ≤2 months prior were enrolled (*N* = 3011) from 250 community, academic, and government sites. No exclusion criteria were applied.

In this analysis patients (excluding those with first progression before informed consent) were categorized into four age groups: <65, 65–74, 75–84, and ≥85 years. The endpoints included time to progression (TTP, excluding all deaths), progression-free survival (PFS), and overall survival (OS). Since shorter OS among patients ≥85 years old was expected, TTP was included to assess the benefit of first-line (1 L) treatment. Statistical analyses are described in Supplemental Methods.

Overall, 3007 patients were included in this analysis (cutoff, February 7, 2019), with a median age of 67 years. Most patients were <75 years (65–74 years: 33%; <65 years: 43%) and 24% were elderly (≥85 years: 4%; 75–84 years: 20%; Supplemental Table 1). More patients in the ≥85-year group had poor prognostic factors at baseline, such as International Staging System (ISS) stage III disease. The proportion of patients with serum creatinine >2.0 mg/dL was similar between groups (20–22%); however, severe and moderate renal impairment was more common in the ≥85-year group. A greater proportion of patients in the ≥85-year group (20%) had a baseline Eastern Cooperative Oncology Group performance status ≥2 versus the <65-year group (9%).

Median time on 1 L treatment decreased with age (<65 years, 17.0 months; ≥85 years, 7.7 months; Supplemental Table 2). Elderly patients (≥75 years old) typically received ≤1 novel agent (83–93%), whereas younger patients (<75 years old) received ≥2 novel agents (33–43%) in 1 L versus elderly pts (8–17%). Fewer elderly patients received triplet regimens as 1 L therapy (18–40%) versus younger patients (56–66%). Stem cell transplant as part of 1 L therapy was more common among younger patients (aged <65 years, 44%; 65–74 years, 25%) versus the elderly (aged 75–84 years, 2%; ≥85 years, 0%). The most common initial therapies in the ≥85-year group were bortezomib–dexamethasone (Vd), lenalidomide–dexamethasone (Rd), lenalidomide–bortezomib–dexamethasone (RVd), and dexamethasone (Supplemental Fig. 1). Younger patients typically received RVd, Vd, cyclophosphamide–bortezomib–dexamethasone, or Rd as initial therapy.

Median PFS shortened with increasing age and was significantly shorter in the ≥85-year group versus all other groups (Fig. 1A). TTP was significantly shorter in the ≥85-year group versus the <65-year group (Fig. 1B); however, there were no significant differences in TTP between ≥85-year and each of the other two age groups. Similar to PFS, OS shortened as age increased, with the ≥85-year group demonstrating significantly shorter median OS than all other groups (Fig. 1C). To isolate the effect of age, outcomes were adjusted for covariates (actual transplant in 1 L, number of comorbidities [<2 versus ≥2], hemoglobin category [<10 or >2 g/dL less than the lower limit of normal versus ≥10 g/dL], calculated ISS stage, novel agents in 1 L, pathological fractures, race, RVd in 1 L, and any triplet therapy in 1 L) that did not result in notable changes in PFS or OS. Moreover, TTP in the adjusted analysis showed no significant differences between any age groups.

**Fig. 1:**
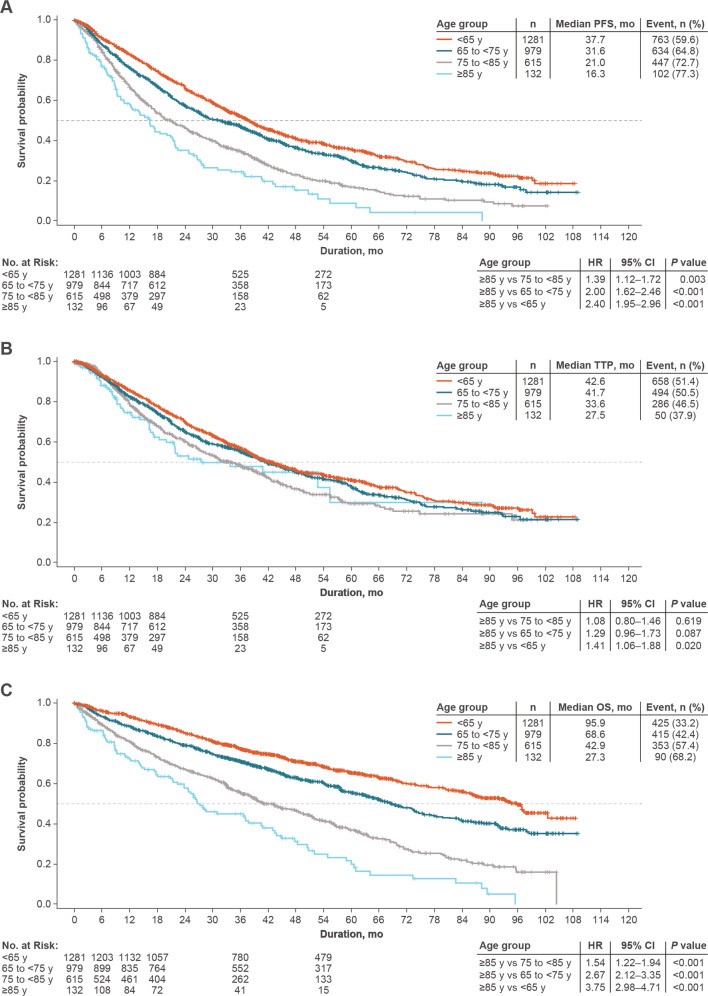
Survival outcomes by age group. **A** Progression-free survival in 1 L. **B** Time to progression in 1 L. **C** Overall survival. *OS* overall survival, *PFS* progression-free survival, *TTP* time to progression.

The mortality rate during 1 L was the highest in the ≥85-year group (46%), mainly attributed to MM progression, and increased in the second- and third-line settings (47% and 54%, respectively; Supplemental Table 2). Similar trends were observed in the younger groups, although the absolute mortality rates in each line of therapy were lower in younger groups. The main cause of death across all age groups and lines of therapy was MM progression. The proportion of deaths due to MM progression was similar in the ≥85-year group (43%) and the <65-year group (40%) in 1 L and remained generally consistent in the ≥85-year group across all lines of therapy. Apart from MM progression, the most common causes of death after 1 L treatment in both the ≥85-year and <65-year groups included cardiac (9% each) and renal (5% each) failure. Ten deaths (1.6%) ≤3 months from the start of MM treatment due to pneumonia or other infections were reported (<65, *n* = 0; 65–74 years, *n* = 3 [1.6%]; 75–84 years, *n* = 4 [1.9%]; ≥85 years, *n* = 3 [5.2%]).

This analysis from the CONNECT MM Registry provides insights into the benefits of active treatment of elderly patients with MM. PFS and OS were significantly shorter in the ≥85-year group versus the younger-age groups; this is expected and consistent with previous reports, as life expectancy is a key factor in OS [4,6,]. Of note, TTP was significantly shorter in the ≥85-year versus the <65-year group, but was similar versus each of the other two age groups, supporting previous data on the potential benefit of active treatment of elderly patients with NDMM [11]. TTP was included as an endpoint in this study, given the competing risk of death, particularly in elderly patients, which was censored in the TTP analysis. Conversely, deaths potentially associated with treatment-related toxicity may be missed when evaluating TTP, which is especially relevant in a vulnerable elderly population [12]. As expected, a higher proportion of patients in the elderly groups died during all reported lines of therapy versus younger groups; however, across all age groups and across each line of therapy, patients remained at the highest risk of dying from their underlying MM, further supporting active treatment of elderly patients with NDMM.

Baseline characteristics were generally comparable across age groups; however, as is common among the elderly, a larger proportion of patients in the ≥85-year group exhibited poor prognostic factors and renal function at baseline versus the three younger groups.

While all age groups received similar 1 L and maintenance regimens, consistent with previous data, the elderly more commonly received doublet (versus triplet) regimens [7]. Differences in treatment strategies, however, did not significantly affect TTP among patients ≥65 years old; further, these data also support previous reports of similar treatment regimens being used across age groups and increased novel agent use in younger patients [4–6].

There are well-known limitations of real-world studies, such as patient registries, including patients not randomized to treatment, the lack of protocol-mandated specific treatments, scheduled clinic visits, and variations in treatment duration and intensity. As there were no scheduled clinic visits, disease-evaluation intervals may have varied between patients. This could affect the assessment of outcome measures (e.g., PFS) and limit comparisons of observational data with clinical trial results. To address these issues, as well as the potential for missing or erroneous data, the prospective nature of the Connect MM^®^ Registry has the ability to query sites for more information on questionable data. Finally, there are barriers to enrollment of elderly patients, even on a nontherapeutic observational registry study, that may have precluded their participation. This may explain the relatively lower proportion of very elderly patients (≥85 years) in this analysis versus the SEER-Medicare database (4% versus 9%) [2], although this could also be explained by the inclusion of patients with smoldering myeloma or patients who deferred therapy in the SEER-Medicare database. Despite these limitations, the Connect MM^®^ Registry allows examination of clinical outcomes in patients treated in a mostly community-based setting, which better reflects real-world populations and clinical practice versus clinical trials.

In summary, the similar regimens and comparable TTP observed across age groups support active treatment, including the use of novel agents and aggressive supportive care for elderly symptomatic patients with MM to improve outcomes and reduce early mortality. To further assess this, barriers to clinical trial enrollment should be revisited, including eligibility criteria and physician, patient, and caregiver perceptions that may discourage trial participation. Additionally, clinical outcomes in elderly patients with NDMM should be further investigated to optimize therapy with novel agents in a manner that balances efficacy and quality of life through assessment of functional status, comorbidities, tolerability, individual therapeutic goals, and disease-related factors that are unique to this patient population.

## Supplementary information

Supplemental Material
