# Supplementary material for: Treatment patterns and outcomes in elderly patients with newly diagnosed multiple myeloma: results from the Connect® MM Registry
Source: Blood Cancer J. 2021 Jul 23;11(7):134. doi: 10.1038/s41408-021-00524-1 (PMC8302734; doi:10.1038/s41408-021-00524-1)
Supplement: Supplementary file 1 — Supplemental Material [file 41408_2021_524_MOESM1_ESM.docx]

**SUPPLEMENT**

**Supplemental Methods**

*Study design and patients*

Patients aged ≥18 years with symptomatic MM (per International Myeloma Working Group criteria) [10], diagnosed ≤2 months prior, were enrolled (*N* = 3011) from 250 community, academic, and government sites. No exclusion criteria were applied. Cohort 1 (*n* = 1493) enrolled from September 2009 to December 2011 and cohort 2 (*n* = 1518) enrolled from December 2012 to April 2016. Informed written consent was required of all patients. Enrollment was competitive to minimize enrollment bias, and all consecutive MM patients presenting to the sites were evaluated for potential enrollment; median time from diagnosis to enrollment was 25 days. The Connect MM^®^ Registry was approved by a central institutional review board (IRB; Advarra, Columbia, MD, USA) or the study site IRB. This Registry is noninterventional, with all medical care was at the discretion of the treating clinician in accordance with the standard clinical practice of their respective site. Patient data are captured in an electronic system at baseline and every 3 months until discontinuation (due to death or patient withdrawal) or end of study (expected in 2024). All patients in the Connect MM^®^ Registry were monitored from time of enrollment until first disease progression (or death), loss to follow-up, or data cutoff.

*Statistical analyses*

Descriptive statistics were used to analyze baseline characteristics and treatment regimens. Study endpoints were compared by age group (≥85 versus <65, 65–74, and 75–84 years), with a focus on initial treatment regimens, treatment outcomes, and use of novel agents in the older age groups (≥85 and 75–84 years). Survival outcomes were analyzed using Kaplan-Meier survival curves and Cox regression with no adjustment. *P* values presented in this paper are nominal with no inferential purpose. PFS was defined as the length of time after the start of MM treatment to progressive disease or death. Overall survival was defined as the length of time from the start of MM treatment to the last date the patient was still alive. TTP analysis was defined similarly to PFS, but deaths were censored in the TTP analysis and not treated as events.

To further isolate the effect of aging, adjusted survival analyses were also conducted, and the comorbidities included in the adjustment were smoking, pacemaker, diabetes, hypertension requiring treatment, venous thromboembolism, HIV, hepatitis, kidney disease, previous malignancy (excluding non-melanoma skin cancer), connective tissue disorder, and other hematologic disorder.

*Data sharing statement*

BMS policy on data sharing may be found at https://www.bms.com/researchers-and-partners/independent-research/data-sharing-request-process.html

**Supplemental Table 1** Baseline characteristics by age group.

| **Characteristic^a^** | **<65 y**  **(*n* = 1281)** | **65–74 y**  **(*n* = 979)** | **75–84 y**  **(*n* = 615)** | **≥85 y**  **(*n* = 132)** |
| --- | --- | --- | --- | --- |
| **Median age (range), y** | 57 (24–64) | 69 (65–74) | 78 (75–84) | 87 (85–94) |
| **Male, n (%)** | 747 (58.3) | 567 (57.9) | 345 (56.1) | 67 (50.8) |
| **Race, n (%)** |  |  |  |  |
| White | 1018 (79.5) | 832 (85.0) | 530 (86.2) | 121 (91.7) |
| Black | 212 (16.5) | 126 (12.9) | 65 (10.6) | 8 (6.1) |
| Other^b^ | 42 (3.3) | 15 (1.5) | 14 (2.3) | 3 (2.3) |
| **del(17p), n (%)** |  |  |  |  |
| Yes | 135 (10.5) | 107 (10.9) | 76 (12.4) | 10 (7.6) |
| No | 774 (60.4) | 606 (61.9) | 363 (59.0) | 67 (50.8) |
| Data not provided | 372 (29.0) | 266 (27.2) | 176 (28.6) | 55 (41.7) |
| **1q gain, n (%)** |  |  |  |  |
| Yes | 57 (4.4) | 60 (6.1) | 29 (4.7) | 3 (2.3) |
| No | 469 (36.6) | 376 (38.4) | 228 (37.1) | 49 (37.1) |
| Data not provided | 755 (58.9) | 543 (55.5) | 358 (58.2) | 80 (60.6) |
| **t(4;14), n (%)** |  |  |  |  |
| Yes | 93 (7.3) | 56 (5.7) | 27 (4.4) | 7 (5.3) |
| No | 776 (60.6) | 625 (63.8) | 384 (62.4) | 67 (50.8) |
| Data not provided | 412 (32.2) | 298 (30.4) | 204 (33.2) | 58 (43.9) |
| **t(11;14), n (%)** |  |  |  |  |
| Yes | 173 (13.5) | 129 (13.2) | 68 (11.1) | 10 (7.6) |
| No | 530 (41.4) | 420 (42.9) | 230 (37.4) | 37 (28.0) |
| Data not provided | 578 (45.1) | 430 (43.9) | 317 (51.5) | 85 (64.4) |
| **t(14;16), n (%)** |  |  |  |  |
| Yes | 40 (3.1) | 28 (2.9) | 23 (3.7) | 5 (3.8) |
| No | 307 (24.0) | 252 (25.7) | 131 (21.3) | 19 (14.4) |
| Data not provided | 934 (72.9) | 699 (71.4) | 461 (75.0) | 108 (81.8) |
| **Hyperdiploidy, n (%)** |  |  |  |  |
| Yes | 98 (7.7) | 74 (7.6) | 46 (7.5) | 8 (6.1) |
| No | 633 (49.4) | 493 (50.4) | 304 (49.4) | 54 (40.9) |
| Data not provided | 550 (42.9) | 412 (42.1) | 265 (43.1) | 70 (53.0) |
| **ECOG PS, n (%)** |  |  |  |  |
| 0–1 | 747 (58.3) | 538 (55.0) | 312 (50.7) | 55 (41.6) |
| 2–3 | 114 (8.9) | 100 (10.2) | 95 (15.4) | 26 (19.7) |
| 4–5^c^ | 1 (0.1) | 2 (0.2) | 0 | 0 |
| Not specified/data not provided^d^ | 419 (32.7) | 339 (34.6) | 208 (33.8) | 51 (38.6) |
| **Calculated ISS stage, n (%)** |  |  |  |  |
| I | 292 (22.8) | 165 (16.9) | 80 (13.0) | 11 (8.3) |
| II | 333 (26.0) | 278 (28.4) | 185 (30.1) | 24 (18.2) |
| III | 321 (25.1) | 281 (28.7) | 178 (28.9) | 56 (42.4) |
| Not specified | 335 (26.2) | 255 (26.0) | 172 (28.0) | 41 (31.1) |
| **Calcium ≥11.5 mg/dl, n (%)** | 127 (9.9) | 84 (8.6) | 43 (7.0) | 15 (11.4) |
| **Serum creatinine > 2.0 mg/dl, n (%)** | 263 (20.5) | 192 (19.6) | 126 (20.5) | 29 (22.0) |
| **Renal function, eGFR, n (%)** |  |  |  |  |
| Normal (>80 ml/min) | 769 (60.0) | 335 (34.2) | 71 (11.5) | 0 |
| Mild impairment  (>50 to ≤80 ml/in) | 283 (22.1) | 376 (38.4) | 247 (40.2) | 25 (18.9) |
| Moderate impairment  (>30 to ≤50 ml/min) | 109 (8.5) | 151 (15.4) | 205 (33.3) | 55 (41.7) |
| Severe impairment  (≤30 ml/min) | 112 (8.7) | 110 (11.2) | 84 (13.7) | 49 (37.1) |
| Data not provided | 8 (0.6) | 7 (0.7) | 8 (1.3) | 3 (2.3) |
| **Hb <10 or >2 g/dl below LLN, n (%)** | 571 (44.6) | 465 (47.5) | 296 (48.1) | 81 (61.4) |
| **MM bone involvement, n (%)** | 1050 (82.0) | 763 (77.9) | 450 (73.2) | 90 (68.2) |
| **1L regimens of interest, n (%)** |  |  |  |  |
| Triplet treatment | 840 (65.6) | 545 (55.7) | 245 (39.8) | 24 (18.2) |
| Stem cell transplant | 548 (43.6) | 235 (24.5) | 13 (2.2) | 0 |
| Alkylator^e^ | 287 (22.4) | 216 (22.1) | 136 (22.1) | 24 (18.2) |
| Novel agent^f^ |  |  |  |  |
| 0 | 50 (3.9) | 48 (4.9) | 56 (9.1) | 29 (22.0) |
| 1 | 677 (52.8) | 604 (61.7) | 452 (73.5) | 93 (70.5) |
| ≥2 | 554 (43.2) | 327 (33.4) | 107 (17.4) | 10 (7.6) |

*1 L* first-line, *ECOG PS* Eastern Cooperative Oncology Group performance status, *eGFR* estimated glomerular filtration rate, *Hb* hemoglobin, *ISS* International Staging System, *LLN* lower limit of normal, *MM* multiple myeloma.

^a^Patients with missing data, data not specified, or unknown data are not included in this table.

^b^Includes American Indian/Alaskan Native, Asian, Pacific Islander, and Other.

^c^One patient in the <65 years group and two patients in the 65 to 74 years group had an ECOG PS 4. There were no patients with ECOG PS 5.

^d^Data were not provided for one patient in the ≥85 years group.

^e^Alkylators are melphalan, cyclophosphamide, and bendamustine.

^f^Novel agents are bortezomib, carfilzomib, lenalidomide, and pomalidomide.

**Supplemental Table 2** Disposition and mortality by age group and line of treatment.

| **Patient Disposition n (%)** | **<65 y** | **65–74 y** | **75–84 y** | **≥85 y** |
| --- | --- | --- | --- | --- |
| **1L** | ***n* = 1257** | ***n* = 959** | ***n* = 596** | ***n* = 126** |
| Ongoing | 182 (14.5) | 131 (13.7) | 45 (7.6) | 3 (2.4) |
| Entered next line | 539 (42.9) | 412 (43.0) | 213 (35.7) | 34 (27.0) |
| Discontinued treatment | 247 (19.7) | 148 (15.4) | 104 (17.5) | 29 (23.0) |
| Median time on 1L treatment, months | 17.0 | 14.1 | 9.7 | 7.7 |
| Deaths | 161 (12.8) | 182 (19.0) | 208 (34.9) | 58 (46.0) |
| Most common (≥10%) known cause of death^a^ |  |  |  |  |
| MM progression | 64 (39.8) | 73 (40.1) | 76 (36.5) | 25 (43.1) |
| Other | 39 (24.2) | 43 (23.6) | 45 (21.6) | 9 (15.5) |
| **2L** | ***n* = 539** | ***n* = 412** | ***n* = 213** | ***n* = 34** |
| Ongoing | 90 (16.7) | 63 (15.3) | 28 (13.2) | 1 (2.9) |
| Entered next line | 289 (53.6) | 215 (52.2) | 102 (47.9) | 13 (38.2) |
| Discontinued treatment | 35 (6.5) | 24 (5.8) | 11 (5.2) | 2 (5.9) |
| Deaths | 95 (17.6) | 92 (22.3) | 67 (31.5) | 16 (47.1) |
| Most common (≥10%) known cause of death**^a^** |  |  |  |  |
| MM progression | 48 (50.5) | 55 (59.8) | 39 (58.2) | 8 (50.0) |
| Pneumonia | 4 (4.2) | 8 (8.7) | 0 | 2 (12.5) |
| Sudden death | 0 | 0 | 0 | 2 (12.5) |
| Other | 19 (20.0) | 10 (10.9) | 9 (13.4) | 2 (12.5) |
| **3L** | ***n* = 289** | ***n* = 215** | ***n* = 102** | ***n* = 13** |
| Ongoing | 36 (12.5) | 34 (15.8) | 13 (12.8) | 1 (7.7) |
| Entered next line | 159 (55.0) | 100 (46.5) | 45 (44.1) | 5 (38.5) |
| Discontinued treatment | 17 (5.9) | 9 (4.2) | 6 (5.9) | 0 (0) |
| Deaths | 68 (23.5) | 62 (28.8) | 38 (37.3) | 7 (53.9) |
| Most common (≥10%) known cause of death**^a^** |  |  |  |  |
| MM progression | 45 (66.2) | 37 (59.7) | 28 (73.7) | 3 (42.9) |
| Renal failure | 3 (4.4) | 2 (3.2) | 0 | 1 (14.3) |
| Pneumonia | 2 (2.9) | 0 | 0 | 1 (14.3) |
| Other | 9 (13.2) | 10 (16.1) | 4 (10.5) | 0 (0) |

*1 L* first-line, *2 L* second-line, *3 L* third-line, *MM* multiple myeloma.

^a^The percent of patients who died of a cause is based upon the number of patients who died in the corresponding line of therapy for that age group. Patients with unknown cause of death are not listed in the table.

**Supplemental Figure 1.** Most common initial therapy (A) and maintenance (B) in 1 L.


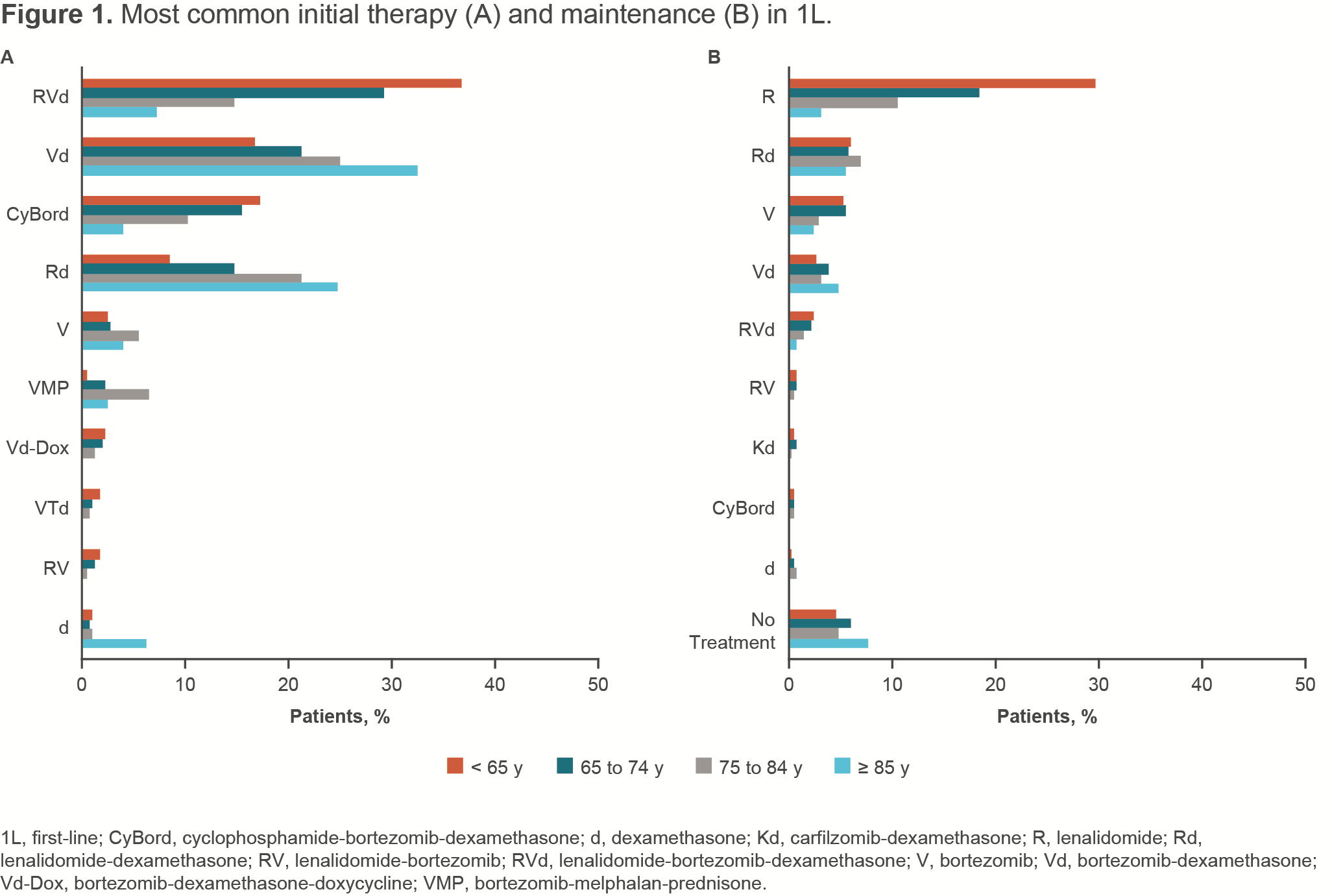


*1 L* first-line, *CyBord* cyclophosphamide-bortezomib-dexamethasone, *d* dexamethasone *Kd* carfilzomib-dexamethasone, *R* lenalidomide, *Rd* lenalidomide-dexamethasone, *RV* lenalidomide-bortezomib, *RVd* lenalidomide-bortezomib-dexamethasone, *V* bortezomib, *Vd* bortezomib-dexamethasone, *Vd-Dox* bortezomib-dexamethasone-doxycycline, *VMP* bortezomib-melphalan-prednisone.
